# The cholinergic contribution to the resting-state functional network in non-demented Parkinson’s disease

**DOI:** 10.1038/s41598-018-26075-3

**Published:** 2018-05-16

**Authors:** Yoonju Lee, Jee Hyun Ham, Jungho Cha, Yeong-Hun Park, Jae Jung Lee, Mun Kyung Sunwoo, Jin Yong Hong, Young H. Sohn, Jong-Min Lee, Phil Hyu Lee

**Affiliations:** 1 Department of Neurology, Yonsei University College of Medicine, Seoul, Korea; 2 Memory and Aging Center, Department of Neurology, University of California, San Francisco, USA; 3 Department of Biomedical Engineering, Hanyang University, Seoul, South Korea; 4 Department of Neurology, Inje University College of Medicine, Ilsan Paik Hospital, Goyang, Korea; 5 Department of Neurology, Bundang Jesaeng General Hospital, Seongnam, Korea; 6 Department of Neurology, Yonsei University Wonju College of Medicine, Wonju, Korea; 7 Severance Biomedical Science Institute, Yonsei University, Seoul, South Korea

## Abstract

The cholinergic system arising from the basal forebrain plays an important role in cognitive performance in Parkinson’s disease (PD). Here, we analyzed cholinergic status-dependent cortical and subcortical resting-state functional connectivity in PD. A total of 61 drug-naïve PD patients were divided into tertiles based on normalized substantia innominata (SI) volumes. We compared the resting-state network from seed region of interest in the caudate, posterior cingulate cortex (PCC), and SI between the lowest (PD-L) and highest tertile (PD-H) groups. Correlation analysis of the functional networks was also performed in all subjects. The functional network analysis showed that PD-L subjects displayed decreased striato-cortical functional connectivity compared with PD-H subjects. Selecting the PCC as a seed, the PD-L patients displayed decreased functional connectivity compared to PD-H patients. Meanwhile, PD-L subjects had significantly increased cortical functional connectivity with the SI compared with PD-H subjects. Correlation analysis revealed that SI volume had a positive correlation with functional connectivity from the right caudate and PCC. The present study demonstrated that PD patients exhibited unique functional connectivity from the caudate and the PCC that may be closely associated with cholinergic status, suggesting an important role for the cholinergic system in PD-associated cognition.

## Introduction

Cognitive impairment, as one of the most disabling non-motor features, is known to occur in the early stages of PD^1^. Although the neural basis of cognitive dysfunction in PD remains unknown, pathological and functional neuroimaging studies suggest that the cholinergic system arising from the basal forebrain plays an important role in cognitive performance. According to a staging study of PD pathology, α-synuclein-positive inclusions in the basal cholinergic forebrain areas simultaneously occur with nigral pathology in the early stage of PD^2^. Similarly, a recent *in vivo* positron emission tomography (PET) study of cerebral acetylcholinesterase demonstrated that cholinergic dysfunction occurs in the early course of PD and is more widespread and profound in the development of PDD^3,4^. Furthermore, our recent volumetric magnetic resonance imaging (MRI) analysis demonstrated that volume loss in the substantia innominata (SI), the major source of cholinergic input, occurs in cognitively normal patients with PD and is most profound in patients with PDD^5^. Moreover, we demonstrated that SI volume is an important factor in predicting the future development of cognitive decline^6^.

Resting-state networks (RSN) are associated with self-oriented mental activity and offer a means of evaluating the status of functional systems within the brain without externally goal-directed cognitive performance^7^. Furthermore, altered RSN is known to be associated with cognitive status in Alzheimer’s disease and dementia with Lewy bodies in addition to PD^8,9^. Therefore, RSN analysis may be helpful in the identification of brain regions functionally coupled with pathological change-dependent processes in neurodegenerative disease. Besides underlying pathological changes, the pattern of RSN is also influenced by several factors, such as various neurochemicals^10^. In terms of cholinergic system, the identification of specific functional network maps that are closely related with cholinergic system never been studied, although this approach would inform our basic understanding about cholinergic influence on cognitive performance in patients with PD. In the present study, we hypothesized that changes in the cholinergic system would influence the functional connectivity pattern in patients with PD. Thus, we performed a comparative analysis of RSN according to the SI volume in non-demented drug naïve PD patients to further elucidate cholinergic system-dependent cortical-subcortical functional networks.

## Results

### Demographic characteristics

The demographic features of the PD and control groups are presented in Table 1. No significant differences in age, gender, disease duration, education duration, UPDRS III score, the K-MMSE score, CCSI score, BDI score, or motor subtype were observed between the PD-L and PD-H groups. In addition, DAT activity in the posterior putamen did not differ significantly between the PD-L and PD-H groups. The mean total brain volume did not differ significantly among the PD-L, PD-H, and control groups (1,292,237.80 vs. 1,282,358.10 vs. 1,261,833.67 vs 1,258,427.86). As expected, the normalized SI volume was smaller in the PD-L group (1.20) compared with the PD-H (1.65) and control groups (1.66, *P* < 0.001). Of 61 patients with PD, 11 patients had normal cognition and 50 had mild cognitive impairment (MCI). The detailed neuropsychological test showed no significant differences in all cognitive tests between the PD-L and PD-H groups (Supplementary Table S1). The number of patients complaining of depression, anxiety, apathy, or sleep disorder did not differ significantly (Supplementary Table S1) between the PD-L and PD-H groups.

**Table 1:** Demographic characteristics of Parkinson’s disease (PD) according to substantia innominate (SI) volume.

	PD N: 61	PD-L N: 20	PD-H N: 21	Control N: 29	*p*-value
Age (year)	69.2 (6.3)	70.9 (4.4)	68.6 (6.1)	71.2 (5.2)	NS
Gender (male, %)	30 (49.2)	11 (55.0)	8 (38.1)	9 (32.1)	NS
Parkinsonism duration, years	2.9 (2.8)	3.1 (2.4)	2.7 (2.0)	—	NS
Education years	9.1 (4.7)	10.0 (5.0)	8.5 (4.8)	10.2 (6.2)	NS
UPDRS III	26.7 (9.5)	25.16 (9.0)	26.1 (8.4)	—	NS
K-MMSE	26.3 (2.3)	26.7 (2.4)	25.8 (2.1)	28.3(1.4)*	0.001
CCSI	6.0 (2.2)	6.1 (1.7)	6.1 (2.4)	10.3 (1.5)*	<0.001
BDI	15.9 (10.1)	14.9 (8.7)	15.5 (10.6)	12.6 (9.5)	NS
PD motor subtype (%)					
Tremor-dominant	32.8	30	28.6	—	NS
PIGD	39.3	35	42.9	—	
Mixed	27.9	35	28.6	—	
DAT activity					
More affected side^†^	0.93 (0.23)	0.93 (0.25)	0.99 (0.28)		NS
Less affected side	1.34 (0.45)	1.47 (0.44)	1.42 (0.57)		NS
Total brain volume, mm^3^	1278531.57 (110503.17)	1292237.80 (120894.66)	1261833.67 (115168.27)	1258427.86 (109816.53)	NS
Normalized SI volume	1.43 (0.21)	1.20(0.08)^‡^	1.65(0.13)	1.66(0.11)	<0.001

Values are expressed as mean (±standard deviation) or number of subjects (%).

PD-S, PD with smaller SI volume group; PD-L, PD with larger SI volume group; UPDRS, unified Parkinson’s disease rating scale; K-MMSE, Korea version of the Mini-Mental State Examination; CCSI, Cross-Cultural Smell Identification; BDI, Beck Depression Inventory; PIGD, Postural instability/gait difficulty; DAT, Dopamine transporter; NS, not significant.

^†^A site where a within-subject DAT activity in the posterior putamen of one hemisphere is lower relative to the other hemisphere.

*Control vs PD-L or PD-H (Bonferroni corrected p-value).

^‡^PD-S vs PD-L or control (Bonferroni corrected p-value).

### Comparative analysis of resting-state striato-cortical connectivity from the seed ROI in the caudate

**Figure 1:**
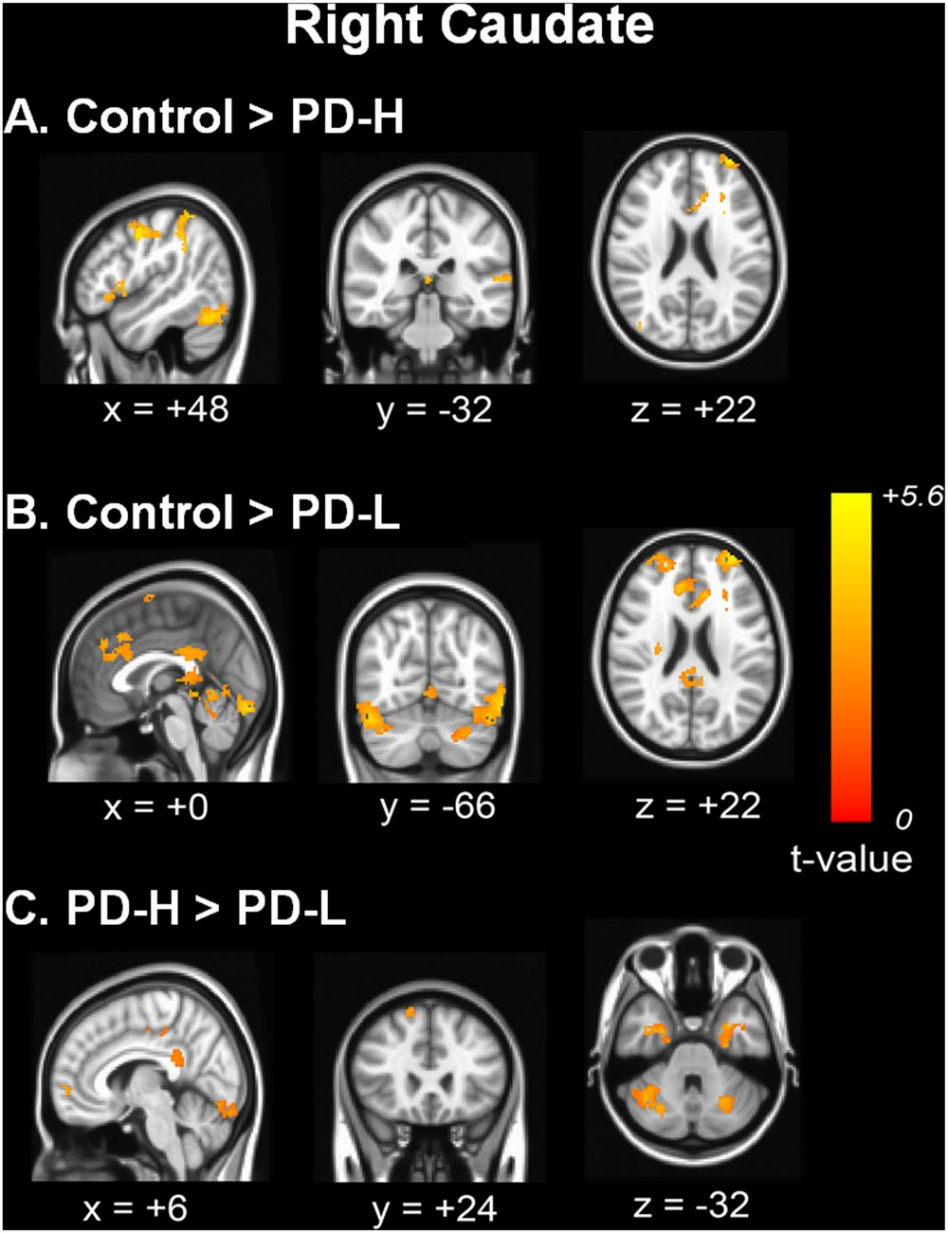
Comparison of functional connectivity from the right caudate nucleus. Functional connectivity in patients with PD-H (**A**) and PD-L (**B**) was compared with that of control subjects. In a direct comparison of the two groups, the PD-L group displayed decreased functional connectivity with the right prefrontal areas, left inferior temporal areas and bilateral cerebellar areas relative to the PD-H group (**C**).

When the right caudate nucleus was selected as a seed, PD-H showed decreased functional connectivity compared with controls in the frontal areas, parietal area, temporal areas, precentral area, posterior cingulate area, and cerebellar areas (Fig. 1A). Similarly, PD-L showed decreased functional connectivity in frontal areas, parietal area, temporal areas, fusiform and posterior cingulate areas, and cerebellar areas relative to controls (Fig. 1B). In a direct comparison of the two groups, PD-L displayed decreased functional connectivity with the prefrontal areas, primary motor areas, posterior cingulate area, and cerebellar areas relative to PD-H (Fig. 1C). No areas of increased cortical functional connectivity were found in the PD-L group relative to the PD-H group. Additionally, using the left caudate as a seed for the RSN analysis, PD-H exhibited decreased striato-cortical functional connectivity compared with normal controls in the postcentral area, parietal area, prefrontal and anterior cingulate areas, temporal areas, fusiform areas, and cerebellar areas (Supplementary Fig. 1A). Similarly, PD-L showed decreased functional connectivity in the postcentral and parietal areas, prefrontal areas, anterior and posterior cingulate, temporal areas, fusiform areas, and cerebellar areas (Supplementary Fig. 1B). In a direct comparison, the PD-L group exhibited decreased striato-cortical functional connectivity in the frontal areas, inferior temporal area, middle and posterior cingulate areas, and cerebellar areas compared with the PD-H group (Supplementary Fig. 1C). No areas of increased cortical RSN were observed in the PD-L group relative to the PD-H group. The anatomical locations of the significant peaks based on the seed ROI in seed region are listed in Supplementary Tables S2 and S3.

### Comparative analysis of cortico-cortical functional connectivity from the seed ROI in the PCC

**Figure 2:**
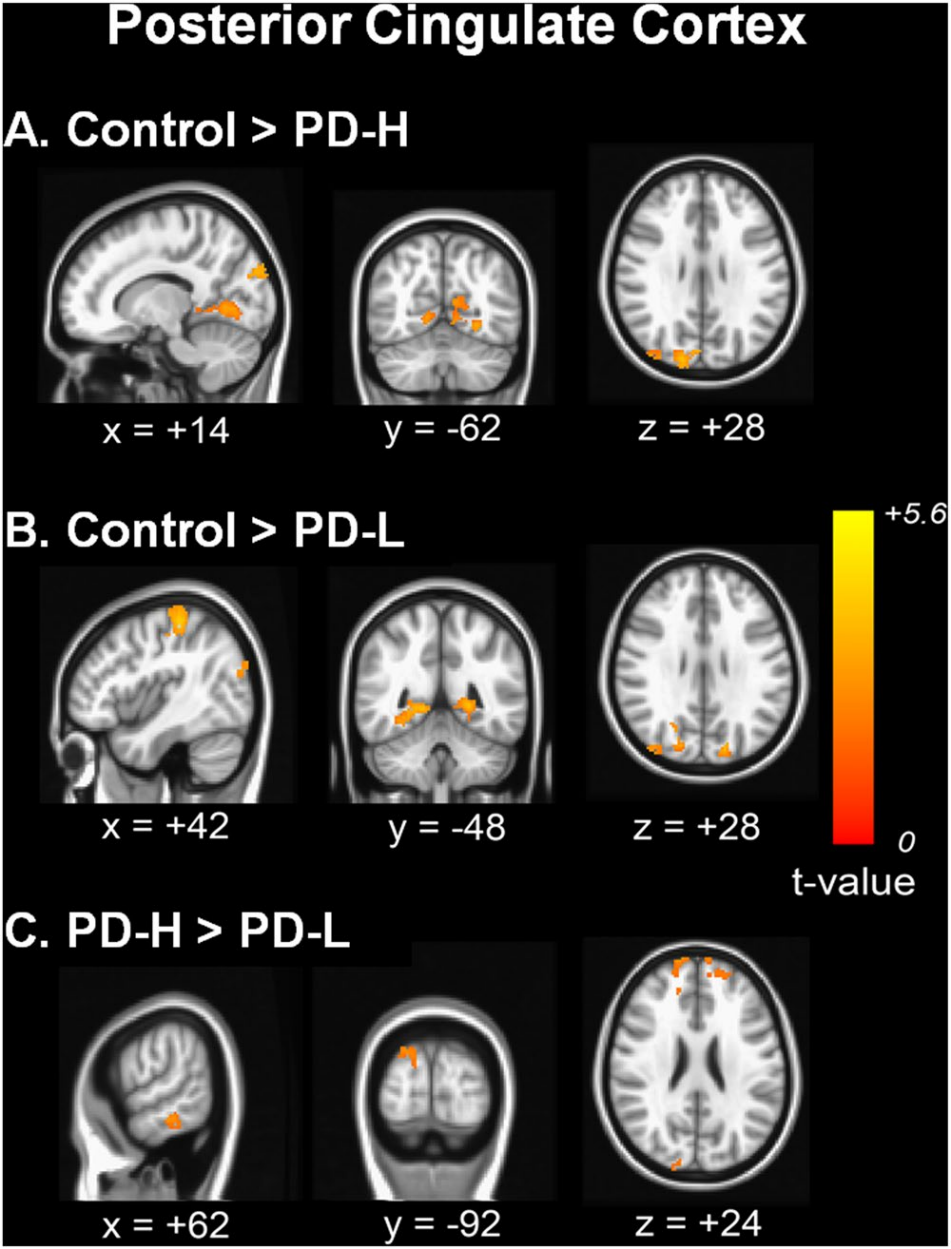
Comparison of cortico-cortical functional connectivity from the posterior cingulate cortex. Functional connectivity in patients with PD-H (**A**) and PD-L (**B**) was compared with that of control subjects. In a direct comparison, the PD group showed decreased functional connectivity with the bilateral prefrontal areas, right inferior temporal areas and posterior cortical areas (**C**).

Compared with the control group, the PD-H group showed decreased functional connectivity with the PCC in the primary motor and sensory areas, precuneus, and occipital areas (Fig. 2A). PD-L showed decreased functional connectivity in the precuneus and occipital areas (Fig. 2B). No areas of increased cortical RSN were observed in the PD-H or PD-L groups compared to the control group. In a direct comparison, the PD-L group displayed decreased functional connectivity with the prefrontal areas, inferior temporal areas, and parietal areas compared with the PD-H group (Fig. 2C). The anatomical locations of the significant peaks are listed in Supplementary Table S4.

### Comparative analysis of cortical and subcortical functional connectivity from the seed ROI in the SI

**Figure 3:**
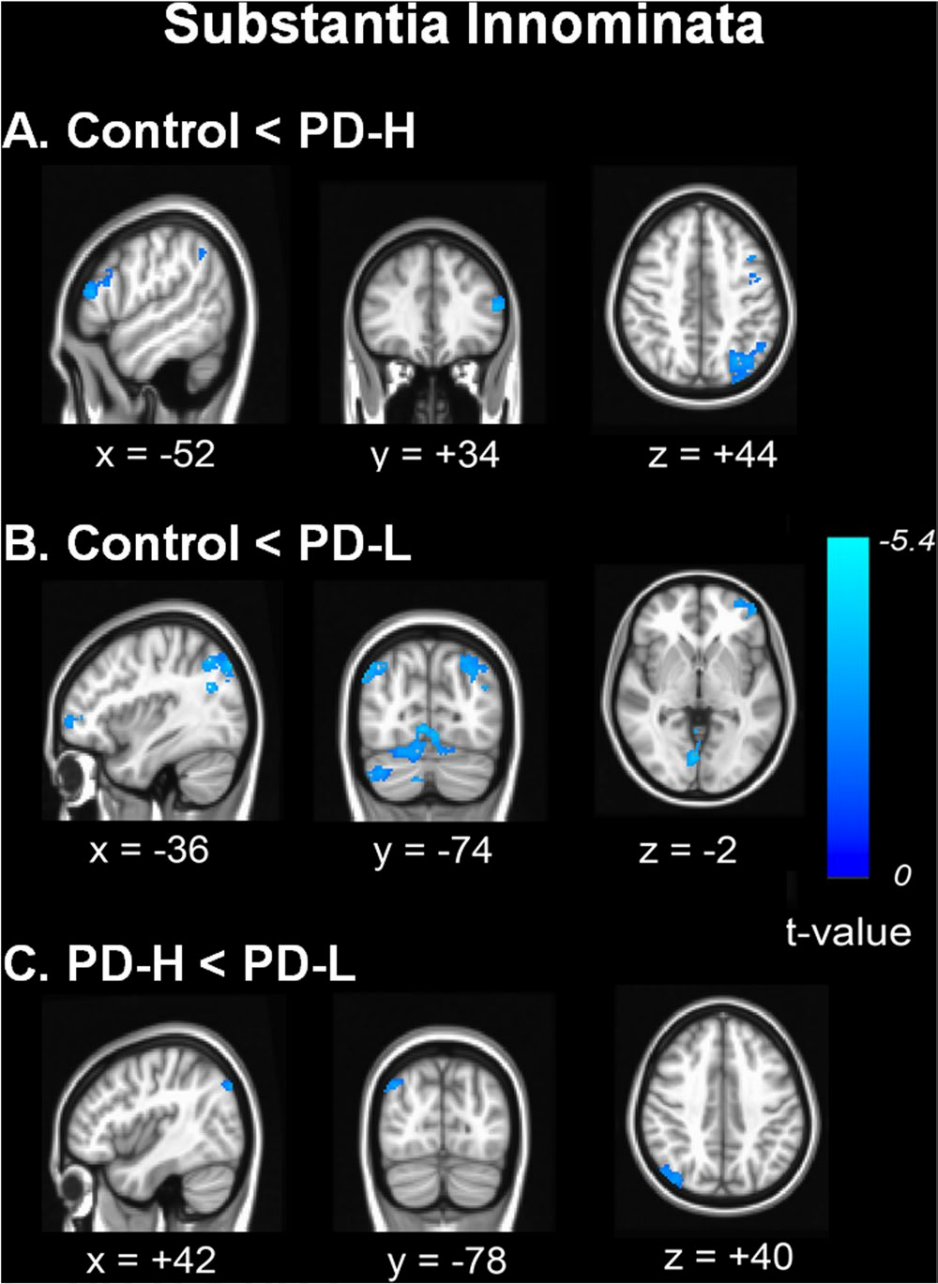
Comparison of cortico-cortical functional connectivity from the substantia innominata. Functional connectivity in patients with PD-H (**A**) and PD-L (**B**) was compared with that of control subjects. In a direct comparison, the PD-L patients had significantly increased cortical functional connectivity mainly in the right temporo-parietal areas compared with PD-H patients (**C**).

Relative to the control group, both the PD-L and PD-H groups had increased cortical functional connectivity with the SI in the prefrontal, posterior cortical, and cerebellar areas; unexpectedly, the area of increased cortical RSN with the SI was more widespread in PD-L than in PD-H (Fig. 3A). No areas of decreased cortical or subcortical connectivity were observed in the PD-L or PD-H groups compared to the control group. In the direct comparison, the PD-L group exhibited increased cortical RSN in the parietal area relative to the PD-H group (Fig. 3B). The anatomical locations of the significant peaks are listed in Supplementary Table S5.

### Correlation analysis of the SI volume and resting state functional connectivity

**Figure 4:**
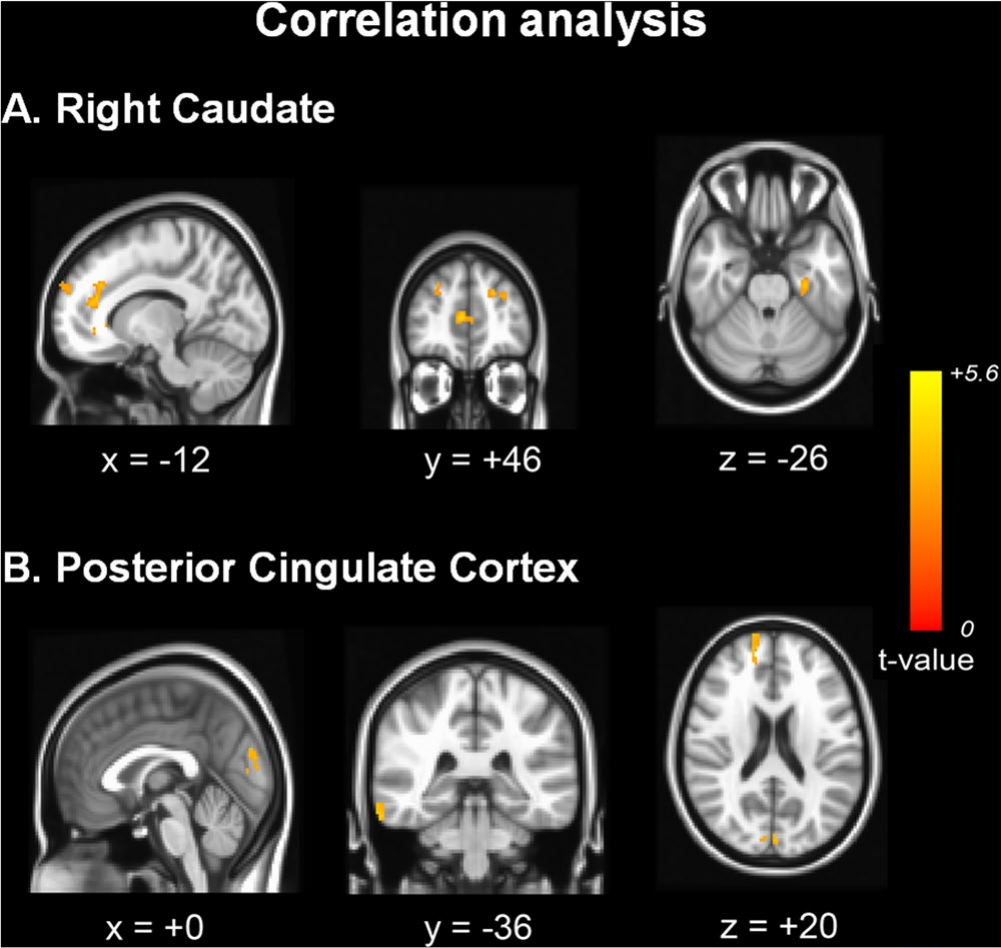
Correlation analysis of the substantia innominate volume and resting state functional connectivity. The SI volume was positively correlated with functional connectivity from the right caudate in the bilateral frontal and left temporal areas (**A**). Using the PCC as a seed, functional connectivity was positively correlated with the SI volume in the right inferior temporal, right prefrontal, and cuneus areas (**B**).

The SI volume was positively correlated with functional network from the right caudate in the frontal and temporal areas (Fig. 4A). However, there were no significant areas in which the SI volume was positively or negatively correlated with cortical RSN from the left caudate nucleus. Using the PCC as a seed for the RSN analysis, the functional connectivity was positively correlated with the SI volume in the inferior temporal, prefrontal, and cuneus areas (Fig. 4B). The anatomical locations of the significant peaks are listed in Supplementary Table S6.

### Correlation analysis of the cognitive total composite score and resting state functional connectivity

Cognitive composite score was positively correlated with functional connectivity from the right caudate in the left parietal area (Supplementary Fig. 2A) and from the left caudate in the bilateral parietal areas (Supplementary Fig. 2B). Using the PCC as a seed, the functional connectivity was positively correlated with cognitive composite score in the bilateral parietal and temporal areas (Supplementary Fig. 2C). Additionally, cognitive composite score had a positive correlation with functional connectivity from SI seed in right prefrontal and bilateral occipital areas (Supplementary Fig. 2D). The anatomical locations of the significant peaks are listed in Supplementary Table S7.

## Discussion

The present study demonstrates that even though cognitive performance is comparable, the RSN pattern would differ depending on the SI volume in non-demented PD subjects. First, the PD-L group showed decreased striato-cortical functional connectivity between the caudate and anterior prefrontal areas, posterior cortical areas and cerebellum relative to the PD-H group. Second, the PD-L group had decreased cortical functional connectivity with the PCC in the bilateral prefrontal, right inferior temporal and posterior cortical areas. Third, the PD-L group had increased cortical functional connectivity with the SI in the right parietal area compared to the PD-H group. Fourth, the SI volume was positively correlated with cortical RSN from the PCC and the caudate. The results of the present study indicate that resting state functional network might be closely coupled with the status of the cholinergic system in non-demented PD.

The SI of the basal forebrain contains the nucleus basalis of Meynert, which is the major source of cholinergic input to the cerebral cortex, and degeneration of the basal forebrain may represent a decline of cholinergic activity in the cerebral cortex. Generally, cholinergic inputs from the basal forebrain play a key role in attention, performance on frontal lobe dependent tests, and memory function through their connections with frontal or basolateral limbic areas^11,12^. More importantly, reduced cholinergic activity in the cortex secondary to degeneration of the SI constitutes an important mechanism underpinning cognitive dysfunction as well as a key player predicting the future development of cognitive decline in PD^5,13^.

The PCC, as a central region in the DMN, has functional connectivity with widespread cortical areas such as the medial prefrontal, dorsolateral prefrontal, inferior parietal, and temporal areas; thus, functional network from the PCC is associated with cognitive processes such as memory, attention, and spatial performance^14^. In patients with PD, a recent study reported that RSN within the DMN was altered and this abnormality was significantly associated with cognitive performance such as memory and visuospatial skills^15^. In addition, distinctive RSN patterns from the PCC seem to be dependent on cognitive status: the functional connectivity of the prefrontal area is attenuated in patients with PDD compared with cognitively normal PD patients^9,16^. In the present study, we found decreased functional connectivity with the PCC in either the PD-L or PD-H group compared to control group. In a direct comparison, the PD-L group had decreased cortical functional connectivity in the bilateral prefrontal, right inferior temporal and posterior cortical areas relative to the PD-H group. Moreover, the SI volume was positively correlated with cortical functional connectivity with the PCC in the right inferior temporal, right prefrontal areas and cuneus areas. Accordingly, the present study provides evidence with regard to RSN, suggesting that PD-L patients have altered cortical RSN with the PCC in the early stages of PD prior to the decline of cognitive performance. This may explain why the status of the cholinergic nucleus acts as a prognostic marker of dementia conversion in PD.

The caudate nucleus, particularly the dorsal caudate, is part of the dorsolateral prefrontal circuit and thus, the caudate is involved in cognitive processes such as executive function, memory and attention. An association between the caudate nucleus and cognitive decline is also evident in patients with PD^6,17^. Regarding RSN between the striatum and cortex, the caudate is functionally coupled with prefrontal, parietotemporal, and cerebellar cortices^18^. Patients with PD show decreased resting-state functional connectivity between the caudate and cortex relative to healthy controls; this is particularly evident in the frontal cortex, involving prefrontal, frontomedial, and orbitofrontal areas^19^, even though this caudate-cortical functional connectivity is not consistent^20^. In the present study, RSN analysis with the caudate seed revealed that PD-L patients had decreased functional connectivity in anterior prefrontal areas, posterior cortical areas and cerebellum compared with PD-H patients. Additionally, correlation analysis demonstrated that cortical functional connectivity with the caudate was positively associated with SI volume in the prefrontal areas. Therefore, the cholinergic nucleus seems to closely modulate cortical functional connectivity with the caudate in patients with PD, further indicating the role of the cholinergic nucleus in PD-related cognitive dysfunction.

Interestingly, the present study also demonstrated that patients with PD had decreased functional connectivity in the cerebellar areas from the caudate nucleus as SI volume became smaller. Functional connectivity between the striatum and cerebellum is well documented in healthy subjects as well as in patients with PD. This striato-cerebellar connectivity appears to be largely influenced by dopaminergic status, as this connectivity is markedly decreased in patients with PD^21^, and exogenous levodopa administration would enhance the striato-cerebellar functional connection in both healthy subjects and PD patients^18,22^. However, in the present study, the decreased striato-cerebellar functional connectivity in PD patients is difficult to explain based on neurochemical changes in dopamine levels, as the severity of parkinsonian motor deficits assessed by the UPDRS motor score and DAT uptake was comparable between patients with PD-L and PD-H. This suggests that cholinergic status may also influence resting-status striato-cerebellar functional connectivity. According to recent studies, this striatocerebellar functional connectivity represents a major subcortical network that influences cognitive processes in addition to movement. Specially, the cerebellum is known to participate in the adaptive modification of behavior and error-based learning^23,24^. Therefore, further research is required to clarify the specific types of cognitive dysfunction modulated by the striatocerebellar functional interconnections via cholinergic intervention.

According to a recent RSN analysis, the SI exhibited functional connectivity with widespread cortical areas, including the prefrontal, orbitofrontal, temporal, and posterior cortical areas, as well as subcortical areas, such as the basal ganglia, thalamus, midbrain, and cerebellum^25^. In the present study, the RSN analysis from the SI seed revealed that the PD-L group had increased cortical functional connectivity in right temporo-parietal areas relative to the PD-H group in contrast to RSN patterns observed in PCC seed. This pattern was similarly observed in the comparative RSN analysis with controls: both PD-L and PD-H patients had increased cortical RSN with the SI, however, the area was more extensive in patients with PD-L than in those with PD-H. It is possible that the enhanced functional connectivity in PD-L patients may be a compensatory process that maintains cholinergic nucleus-dependent cognitive performance in PD. Nevertheless, a further study of serial changes in RSN patterns from the SI after cholinergic modulation is required to uncover this issue.

There are several limitations of this study. First, even though this study enrolled drug-naïve de novo PD patients to exclude as much of the effect of dopaminergic medications on RSN as possible, the number of subjects in each group was relatively small to draw firm conclusions. Second, several factors that are commonly encountered in patients with PD such as olfactory dysfunction or depression may influence the pattern of RSN^26,27^. The CCSI and BDI scores were comparable between the groups, however, we could not completely exclude the impact of these factors on RSN patterns. Finally, the cortical and subcortical seeds used in the present study may not represent a complete functional connectivity analysis, and further study using other imaging analytic tools is required to explain the functional network maps.

In summary, the present study demonstrated that patients with PD exhibited unique resting-state functional connectivity from the caudate and the PCC that may be closely associated with cholinergic status. The identification of cholinergic status-dependent RSN may advance the current understanding of PD pathophysiology with respect to cognition, which may have an important impact on future trials of cognitive modulation in non-demented stage of PD.

## Methods

### Subjects

Sixty-one patients with non-demented PD and 29 normal healthy controls were sequentially enrolled between September 2011 and December 2014 from a movement clinic at a university hospital. All patients fulfilled the criteria for PD as proposed by the UK PD Society Brain Bank^28^ and exhibited reduced dopamine transporter uptake in the posterior putamen on a [^18^F] FP-CIT PET scan. Parkinsonian motor symptoms were evaluated by the Unified PD Rating Scale Part III (UPDRS- III). We calculated a “tremor score” and a “postural instability/gait difficulty (PIGD) score” for each patient as described previously^29^ and classified patients into three clinical subtypes: tremor dominant, PIGD, and mixed. Olfactory function was assessed using a cross-cultural smell identification (CCSI) test^30^, which has been widely used to evaluate the odor identification of PD patients in many countries^31^. Exclusion criteria included presence of probable PD dementia^32^, obvious tremor that would cause motion related artifact for functional MRI analysis, structural lesions on brain MRI, Parkinsonian plus syndromes, a history of using drugs that can cause parkinsonism (antipsychotics, gastrointestinal kinetics, antiepileptic drugs, or L-type calcium channel blockers), or depression (a score >21 on the Beck Depression Inventory (BDI)). The Seoul Neuropsychological Screening Battery was used to evaluate detailed cognitive performance. This battery includes the following scored tests: Digit Span test (forward and backward), the Korean version of the Boston Naming Test, Rey Complex Figure Test (copying, immediate and 20-min delayed recall, and recognition), drawing an interlocking pentagon, Seoul Verbal Learning Test (immediate recall, 20-min delayed recall, and recognition), phonemic and semantic Controlled Oral Word Association Test, go-no-go test and contrasting program, and Stroop Test (word and color reading of 112 items during a 2-min period)^6^. A diagnosis of PD-MCI was made based on the criteria suggested by the Movement Disorder Society Task Force guidelines^33^. Psychopathology (depression, anxiety, apathy or sleep disorder) was assessed using the Korean version of neuropsychiatric inventory performed at the first visit^34^. The control group had no history of neurological disorders and no cognitive complaints with a minimal score on the Korean version of the Mini Mental State Examination (K-MMSE) of 27.

### MR imaging analysis

#### Image acquisition

The study subjects performed functional MRI scanning using a 3.0 Tesla MRI scanner (Achieva, Philips Medical System, Best, Netherlands). A high-resolution T1-weighted MRI volume data set was obtained from all subjects using 3D T1-TFE sequence configured with the following acquisition parameters: axial acquisition with a 224 × 256 matrix; 256 × 256 reconstructed matrix with 182 slices in the coronal plane; 1-mm-thick sections; 220-mm field of view; 0.98 × 0.98 × 1.2 mm^3^ voxels; TE, 4.6 ms; TR, 9.6 ms; flip angle, 8°; slice gap, 0 mm. To obtain T2*-weighted single shot echo planar imaging sequences, each participant was axially scanned using the following parameters: voxel size, 2.8 × 2.8 × 3.0 mm^3^; slice number, 31 (interleaved); matrix, 80 × 80; slice thickness, 3.0 mm; gap, 1.0 mm; repetition time (TR), 2,000 ms; echo time (TE), 30 ms; flip angle = 90°; and field of view, 220 mm. Each 330-sec scan produced 165 functional MRI images, which is known to be enough to assess resting- state functional connectivity^35^ and to obtain low-frequency oscillations for resting-state functional connectivity. During the functional MR imaging, subjects were instructed to remain awake with their eyes closed and to not move or focus on a specific thought.

#### Volumetric determination of the SI

The analysis was done using Statistical Parametric Mapping software (SPM8, Wellcome Department of Imaging Neuroscience, London, UK, available at http://www.fil.ion.ucl.ac.uk/spm). Each structural MRI was bias-corrected, segmented into SPM default tissue probability maps, and then normalized with VBM8 DARTEL templates for registration to MNI space, using linear (12-parameter affine) and non-linear transformations within a unified model. According to the previous study^36^, the region of interest (ROI) of SI was defined one for the left and the other for the right hemisphere, based on the location of the anterior commissure, which forms the boundary of the superior part of the end of the anterior third of the substantia innominata. The ROI extended 25 mm lateral from the midline, 13 mm ventral from the superior edge of the anterior commissure at the midline, and 3 mm anterior and 9 mm posterior from the middle of the anterior commissure. The masks were created using the WFU PickAtlas 2.4 software36, and volumetry of grey matter within selected ROI was done automatically. To correct for individual brain size, volumes were normalized by dividing with total brain volume derived from the masks covering entire brain. Normalized SI volume was defined by the following formula: total SI volume (mm^3^)/total brain volume (mm^3^) X 10,000. Based on the distribution, the normalized SI volumes were categorized into tertile groups; PD with the lowest SI volume (PD-L, n = 20), PD with an intermediate SI volume (n = 20), and PD with the highest SI volume (PD-H, n = 21).

#### Preprocessing of resting-state functional MRI data

Functional brain MRI analyses were performed following a previously described method^37^. Preprocessing of resting-state fMRI data was conducted using the Analysis of Functional NeuroImages, (http://afni.nimh.nih.gov/afni) software^38^. For stabilization of the magnetic field, the initial five volumes from each functional image were eliminated. The eliminated images were de-spiked, and then applied the slice timing and head motion corrections. At the motion-correction corrections, displacement owing to head motion effect was estimated using the head motion-correction parameters of the x, y, and z translations and the Yaw, Pitch and Roll rotation. For every subject, the estimated displacement due to head motion was satisfied the condition: between successive time-series volumes <1 mm and any of the three translation directions <2 mm, or maximum rotation around any of the axes less than 2.0°. Then, the data were corrected using the anatomy-based correlation corrections method known as ANATICOR^39^. The ANATICOR is regression model for removing the not interest signal by the following: (1) six parameters acquired by correction of head motion, (2) signal from the eroded large ventricle mask, and (3) signal from the local white matter erosion mask (r = 15 mm). Subsequently, a band-pass filter (0.009 < f < 0.08) to remove physiological noise was temporally applied to the regressed data. The filtered data were masked out using the gray matter mask to remove the unwanted BOLD or other physiological signals occurring on account of large draining vessels. The spatial smoothing with Gaussian kernel of 6-mm full width at half maximum (FWHM) was conducted to the mask processed functional data. The spatial normalization to a standard MNI152 template was performed to the blurred functional data by the resampling with an isotropic voxel size of 2 mm. The head coil and hardware artifacts were modeled with signal from the eroded local white matter and signal from the eroded large ventricle masks. The white matter mask and large ventricle masks were classified from the registered and non-uniformity corrected T1-weighted images using an advanced neural-net classifier^40^ as well as non-linear warping algorithm^41^. For co-registration between the anatomical T1-weighted image and the functional echo planar image, the affine transform was used with Local Pearson Correlation cost function^42^. Also, all masks were transformed using by same transform matrix into functional echo planar image space. The white matter mask and the large ventricle masks were eroded by one voxel to minimize the partial volume effects.

#### Functional connectivity analysis

To analyze functional connectivity, four ROIs were defined as the bilateral caudate, posterior cingulate cortex (PCC), and SI. First, the caudate was selected to exam functional connectivity between the basal ganglia and cerebral cortices^43^. The caudate was defined based on the automated anatomical labeling template^44^. Second, the PCC, which was highly associated with the default mode network (DMN) in the resting-state functional MRI analysis, was selected. The PCC has been widely described in recent years in PD^20,45^, because the network involving this region plays a key role in many cognitive functions such as memory, attention and problems maintaining the balance between internal and external thought^46^, which are important cognitive domains in PD. Additionally, it was the only node in the DMN that directly interacted with virtually all other nodes^47^. This region was described as a 6-mm radius sphere at the peak (x/y/z = 0/−52/30) using a voxel mask^48^, because this represents the main functional connectivity hub of the human^49^. The PCC ROI seed used for the DMN approach in the present study was based on the previous studies^48–50^. Finally, as the major source of cholinergic input to the cerebral cortex and an important neuroanatomical correlate for cognitive performance in PD, we selected the bilateral SI as a ROI. The time course for data in every ROI was averaged as reference signals. Then, the Pearson’s correlation coefficient was computed for each individual subject with averaged reference signals. The correlation coefficient was converted using Fisher’s z transformation.

#### Group comparisons

To assess the differences in functional connectivity among the PD-L, PD-H, and control groups, an analysis of covariance (ANCOVA) was employed using covariates as age and gender. In this stage, Monte Carlo simulations were performed to adjust for Type I errors (parameters: individual voxel *P* = 0.02, simulation = 10,000 times iteratively, 6-mm FWHM Gaussian filter width with a whole-brain mask) with the AlphaSim program, which provided an estimate of the overall significant level in the AFNI to determine a probability of the false positive detection from a frequency count of cluster size. This was achieved for a variety of probability thresholds combinations for each voxel and cluster size threshold. The corrected significant value was obtained as level of *P*α < 0.05 (uncorrected individual voxel height threshold of *P* < 0.02, F > 4.157 with a minimum cluster size of 1,672 mm^3^). Afterward, significant areas determined by ANCOVA were applied to *Post hoc* two sample *t-test* between pairs of groups at *P*α < 0.05 (corrected significant level).

#### Relationship between functional connectivity and SI value analysis

To investigate the association between resting state functional connectivity from the caudate or PCC seeds and the SI volume, a multiple regression analysis was performed in all PD subjects (n = 61) with age, gender, and education duration entered as covariates. The dependent variable was z-scores of functional connectivity maps and the independent variable was normalized SI value. The statistical maps were corrected for multiple comparisons to a significance level of *P*α < 0.05.

#### Relationship between functional connectivity and total cognitive composite score

A multiple regression analysis was performed between total cognitive composite score and resting state functional connectivity from the caudate, PCC or SI seed in all PD subjects with age, gender, and education duration entered as covariates. The statistical values were same as above.

### Image acquisition and quantitative analysis of the ^18^F-FP-CIT PET data

Quantitative analyses were performed following a modified version of a previously described procedure^51^. Image processing was performed using SPM8 (Wellcome Department of Imaging Neuroscience, Institute of Neurology, UCL, London, UK) under Matlab 6.5.1 for Windows (Math Works, Natick, MA, USA) and MRIcro version 1.37 (Chris Rorden, Columbia, SC, USA). Quantitative analyses were based on the volumes of interest (VOIs), which were defined based on a template in the standard space. All reconstructed PET images were spatially normalized to the Talairach space using a standard ^18^F-FP-CIT PET template which was made using ^18^F-FP-CIT PET and T1 MR images of 13 normal controls to remove inter-subject anatomical variability. The VOIs of bilateral striatal subregions and one occipital VOI were drawn on a co-registered spatially normalized single T1 MR and ^18^F-FP-CIT PET template image. The striatum was divided into the caudate, ventral striatum, anterior putamen, and posterior putamen. Dopamine transporter (DAT) activity was calculated by the non-displaceable binding potential, which was defined as follows: (mean standardized uptake value of the striatal subregion VOI – mean standardized uptake value of the occipital VOI)/mean standardized uptake value of the occipital VOI^52^. Of the VOIs, DAT activity in the posterior putamen was selected because this area is the most severely affected in early stage of PD^53^, and thus it likely reflects dopamine levels in the striata of individual patients.

### Statistical analysis

All data are expressed as means (SDs). The analysis of variance test or the Chi Square test for continuous and categorical variables, respectively. Post hoc analyses were performed using a Bonferroni test to correct for multiple comparisons. Statistical analyses were performed using commercially available software (SPSS, ver.20.0), and a two tailed *P* < 0.05 was considered significant.

### Ethical approval

This study was approved by the Yonsei University Severance Hospital ethical standards committee on human experimentation for experiments using human subjects and was therefore performed in accordance with the ethical standards laid down in the 1964 Declaration of Helsinki.

### Informed consent

Because this study was a retrospective analysis of medical data, we were not required to obtain patient consent.

## Electronic supplementary material

Supplementary information
