# Supplementary material for: The cholinergic contribution to the resting-state functional network in non-demented Parkinson’s disease
Source: Sci Rep. 2018 May 16;8:7683. doi: 10.1038/s41598-018-26075-3 (PMC5955917; doi:10.1038/s41598-018-26075-3)
Supplement: Supplementary file 1 — Supplementary information [file 41598_2018_26075_MOESM1_ESM.doc]

ONLINE SUPPLEMENT

**The cholinergic contribution to the resting-state functional network in non-demented Parkinson’s disease**

Yoonju Lee, Jee Hyun Ham, Jungho Cha, Yeong-Hun Park, Jae Jung Lee, Mun Kyung Sunwoo, Jin Yong Hong, Young H. Sohn, Jong-Min Lee, & Phil Hyu Lee

**Supplementary Fig. 1. Comparison of functional connectivity from the left caudate nucleus.**

Functional connectivity in patients with PD-H (A) and PD-L (B) was compared with that of control subjects. In a direct comparison of the two groups, the PD-L group displayed decreased functional connectivity with the right prefrontal areas, left inferior temporal areas and bilateral cerebellar areas relative to the PD-H group (C).


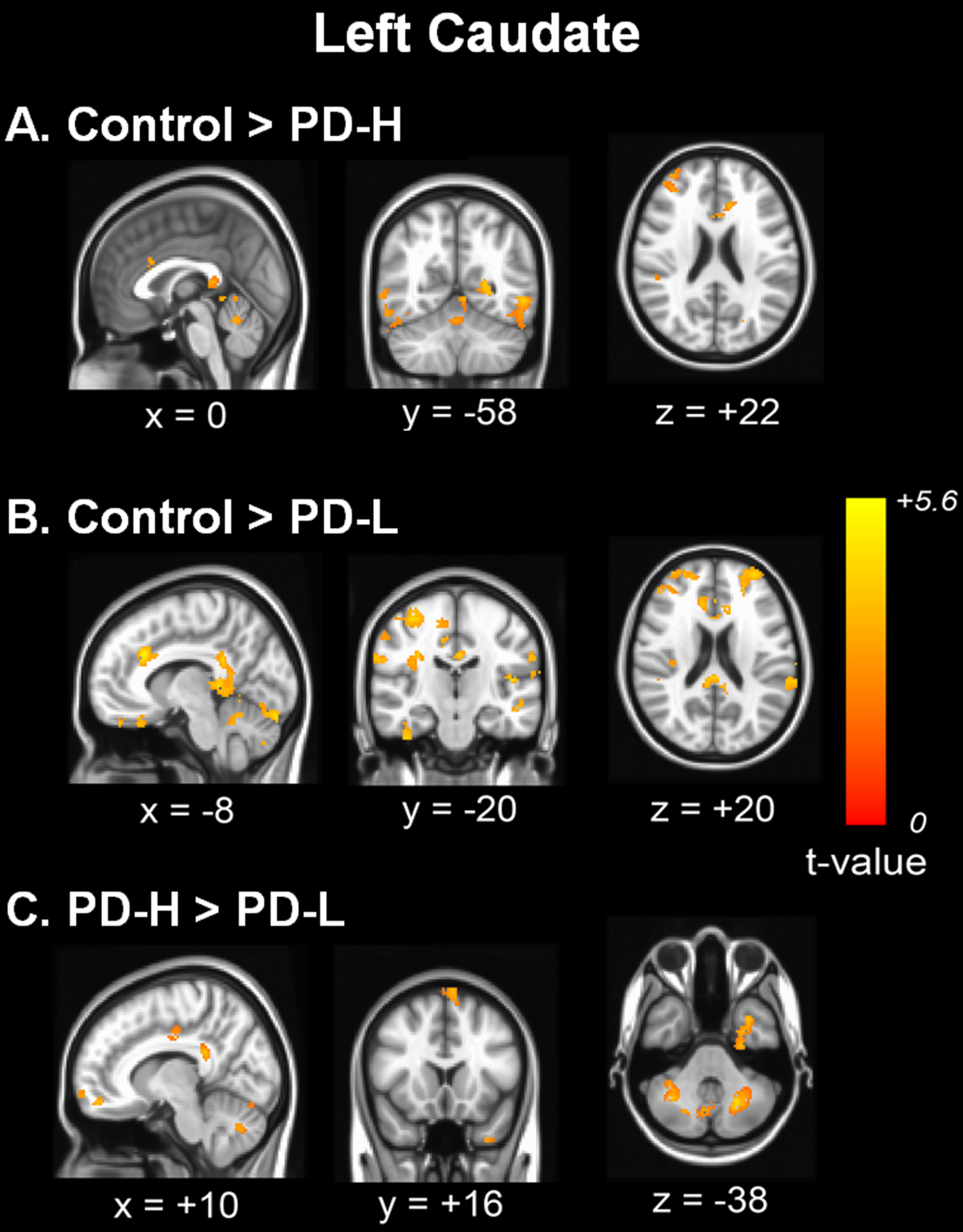


**Supplementary Fig. 2. Correlation analysis between the cognitive total composite score and resting state functional connectivity.** The cognitive total composite score was positively correlated with functional connectivity from the right caudate in the left parietal areas (A) and from the left caudate in the bilateral parietal areas (B). Additionally, functional connectivity was positively correlated with the composite score from the PCC seed in the bilateral parietal and temporal areas (C), and from the SI seed in the right prefrontal and bilateral occipital areas (D).

**
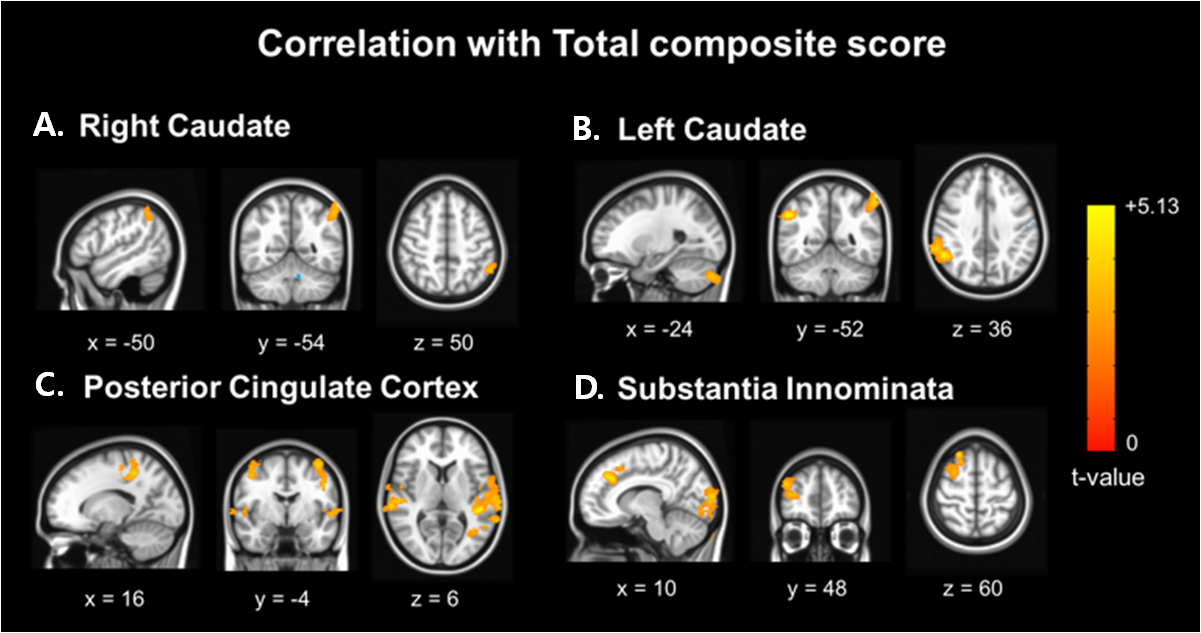
**

**Supplementary Table 1.** Neuropsychological and psychopathology data in Parkinson’s disease (PD) according to substantia innominate (SI) volume

|  | PD-L  (n=20) | PD-H  (n=21) | *p-value* |
| --- | --- | --- | --- |
| Neuropsychological test |  |  |  |
| **Attention** | 45.5 ± 8.7 | 48.2 ± 8.1 | 0.220* |
| Digit span (forward) | 6.0 ± 1.2 | 7.2 ± 1.7 | 0.212 |
| Digit span (backward) | 3.1 ± 0.7 | 3.8 ± 0.9 | 0.134 |
| Word Stroop test | 111.2 ± 9.0 | 104.4 ± 18.7 | 0.313 |
| Color Stroop test | 61.0 ± 33.7 | 77.4 ± 23.4 | 0.162 |
| **Language and related function** | 28.6 ± 4.4 | 27.8 ± 5.4 | 0.490* |
| K-BNT | 43.1 ± 8.6 | 40.5 ± 10.7 | 0.380 |
| Repetition | 14.1 ± 0.9 | 14.2 ± 1.0 | 0.367 |
| **Visuospatial function** | 18.1 ± 2.9* | 18.0 ± 4.1* | 0.918* |
| RCFT | 30.4 ± 5.9 | 30.3 ± 7.3 | 0.884 |
| Pentagon drawing test | 5.9 ± 0.4 | 5.6 ± 1.0 | 0.804 |
| **Verbal memory function (SVLT)** | 13.1 ± 1.8 | 13.6 ± 2.8 | 0.722* |
| Immediate recall | 15.4 ± 3.9 | 16.8 ± 6.0 | 0.649 |
| Delayed recall | 3.7 ± 1.1 | 4.3 ± 2.8 | 0.590 |
| Recognition | 20.3 ± 1.7 | 19.8 ± 1.9 | 0.355 |
| **Visual memory function (RCFT)** | 12.2 ± 5.4 | 13.5 ± 4.4 | 0.478* |
| Immediate recall | 8.6 ± 7.7 | 10.6 ± 6.5 | 0.476 |
| Delayed recall | 8.9 ± 7.3 | 8.9 ± 6.2 | 0.835 |
| Recognition | 19.0 ± 2.0 | 20.4 ± 2.0 | 0.296 |
| **Frontal executive function** | 16.5 ± 1.9 | 15.6 ± 2.4 | 0.612* |
| Contrasting program | 19.6 ± 1.1 | 19.4 ± 1.4 | 0.985 |
| Go-no-go test | 18.9 ± 1.8 | 19.1 ± 1.3 | 0.262 |
| Phonemic generative naming | 17.3 ± 2.9 | 12.2 ± 7.2 | 0.112 |
| COWAT (Animal) | 13.7 ± 2.2 | 14.0 ± 3.0 | 0.695 |
| COWAT (Supermarket) | 12.9 ± 4.7 | 13.3 ± 4.4 | 0.491 |
| Psychopathology (%) |  |  |  |
| Depression | 8 (40) | 9 (43) | > 0.999 |
| Anxiety | 10 (50) | 9 (43) | 0.758 |
| Apathy | 11 (55) | 7 (33) | 0.215 |
| Sleep disorder | 10 (50) | 8 (38) | 0.443 |

Values are expressed as mean ± SD.

Data are adjusted for age, sex, and years of education.

*Group comparison with composite scores for cognitive domains.

PD-S, PD with smaller SI volume group, PD-L, PD with larger SI volume group, K-BNT: Korean version of Boston Naming Test, RCFT: Rey Complex Figure Test, SVLT: Seoul Verbal Learning Test, COWAT: Controlled Oral Word Association Test.

**Supplementary Table 2**. Clusters displaying significantly different resting-state functional connectivity from seed region of interest in the right caudate.

|  | **Brain Regions** | **Side** | **Stereotaxic Coordinates (mm)** | | |  | **Maximum** | **Voxels** |
| --- | --- | --- | --- | --- | --- | --- | --- | --- |
|  |  |  | **x** | **y** | **z** |  | **t** |  |
| Control > PD-H | Fusiform Gyrus | R | 50 | -60 | -22 |  | 3.53 | 1061 |
|  | Fusiform Gyrus | L | -46 | -70 | -22 |  | 4.13 | 826 |
|  | Posterior Cingulate Gyrus | L | 0 | -36 | 10 |  | 4.09 | 589 |
|  | Superior Parietal Lobule | R | 48 | -48 | 70 |  | 4.68 | 421 |
|  | Culmen | L | -28 | -46 | -32 |  | 3.31 | 402 |
|  | Middle Temporal Gyrus | R | 24 | 20 | 32 |  | 4.01 | 392 |
|  | Middle Frontal Gyrus | R | 50 | 0 | 42 |  | 4.12 | 374 |
|  | Superior Temporal Gyrus | L | -56 | 22 | -32 |  | 4.22 | 340 |
|  | Cerebellar Tonsil | R | 32 | -40 | -40 |  | 3.25 | 310 |
|  | Superior Frontal Gyrus | L | -32 | 64 | 22 |  | 4.05 | 240 |
| Control < PD-L | Cerebellar Tonsil | R | 32 | -50 | -38 |  | 5.58 | 8461 |
|  | Superior Frontal Gyrus | L | -32 | 58 | 20 |  | 5.28 | 1834 |
|  | Posterior Cingulate | L | -8 | -48 | 8 |  | 4.68 | 1526 |
|  | Inferior Parietal Lobule | R | 34 | -24 | 34 |  | 4.42 | 1221 |
|  | Middle Temporal Gyrus | L | -36 | 0 | -38 |  | 4.83 | 946 |
|  | Precentral Gyrus | R | 16 | -32 | 58 |  | 4.43 | 799 |
|  | Uncus | R | 26 | -4 | -32 |  | 4.40 | 676 |
|  | Superior Frontal Gyrus | R | 42 | 60 | 26 |  | 3.83 | 419 |
|  | Superior Temporal Gyrus | L | -46 | -18 | 10 |  | 4.89 | 393 |
|  | Parahippocampal Gyrus | R | 26 | -52 | -4 |  | 3.46 | 293 |
|  | Inferior Frontal Gyrus | R | 48 | 22 | -4 |  | 4.14 | 241 |
|  | Orbital Gyrus | L | -16 | 30 | -30 |  | 5.28 | 240 |
| PD-H > PD-L | Tuber | R | 40 | -58 | -36 |  | 4.31 | 512 |
|  | Superior Frontal Gyrus | R | 16 | 62 | -10 |  | 5.43 | 495 |
|  | Middle Temporal Gyrus | L | -32 | 6 | -42 |  | 4.78 | 443 |
|  | Middle Temporal Gyrus | R | 38 | -2 | -34 |  | 3.95 | 365 |
|  | Pyramis | L | -22 | -62 | -38 |  | 4.65 | 311 |
|  | Precentral Gyrus | R | 16 | -32 | 58 |  | 4.13 | 282 |
|  | Precentral Gyrus | L | -28 | -32 | 64 |  | 3.29 | 268 |
|  | Posterior Cingulate | R | 10 | -38 | 24 |  | 3.70 | 247 |
|  | Medial Frontal Gyrus | L | -8 | -6 | 58 |  | 3.82 | 230 |

PD with the lowest SI volume group=PD-L; PD with the highest SI volume group=PD-H; R = right; L = left

**Supplementary Table 3**. Clusters displaying significantly different resting-state functional connectivity from seed region of interest in the left caudate

|  | **Brain Regions** | **Side** | **Stereotaxic Coordinates (mm)** | | |  | **Maximum** | **Voxels** |
| --- | --- | --- | --- | --- | --- | --- | --- | --- |
|  |  |  | **x** | **y** | **z** |  | **t** |  |
| Control > PD-H | Postcentral Gyrus | R | 46 | -48 | 70 |  | 5.39 | 3270 |
|  | Culmen | R | 2 | -44 | -4 |  | 4.56 | 1335 |
|  | Tuber | R | 56 | -64 | -34 |  | 3.56 | 970 |
|  | Fusiform Gyrus | L | -46 | -70 | -22 |  | 4.13 | 812 |
|  | Superior Parietal Lobule | R | 24 | -70 | 48 |  | 3.99 | 563 |
|  | Superior Frontal Gyrus | R | 42 | 60 | 26 |  | 4.41 | 468 |
|  | Anterior Cingulate | R | 20 | 28 | 26 |  | 3.60 | 365 |
|  | Fusiform Gyrus | R | 38 | -14 | -26 |  | 4.55 | 311 |
|  | Middle Temporal Gyrus | L | -44 | 0 | -26 |  | 3.29 | 291 |
| Control > PD-L | Postcentral Gyrus and Superior Parietal Lobule | R | 18 | -30 | 56 |  | 4.83 | 4092 |
|  | Cerebellar Tonsil | L | -22 | -62 | -40 |  | 4.90 | 3062 |
|  | Fusiform Gyrus | R | 50 | -48 | -24 |  | 4.90 | 2150 |
|  | Posterior Cingulate | L | -10 | -48 | 8 |  | 4.99 | 1814 |
|  | Inferior Temporal Gyrus | L | -32 | -4 | -42 |  | 5.2 | 1122 |
|  | Middle Temporal Gyrus | R | 44 | 48 | 30 |  | 4.43 | 650 |
|  | Uncus | R | 30 | -16 | -40 |  | 4.95 | 642 |
|  | Anterior Cingulate | L | -10 | 24 | 28 |  | 4.98 | 629 |
|  | Inferior Frontal Gyrus | R | 18 | 30 | -22 |  | 4.36 | 530 |
|  | Inferior Frontal Gyrus | R | 48 | 24 | -4 |  | 4.28 | 382 |
|  | Inferior Frontal Gyrus | L | -14 | 30 | -24 |  | 4.19 | 368 |
|  | Middle Temporal Gyrus | L | -46 | -28 | -10 |  | 4.96 | 364 |
|  | Superior Frontal Gyrus | L | -32 | 56 | 20 |  | 4.16 | 341 |
|  | Inferior Temporal Gyrus | R | 64 | 0 | -36 |  | 4.23 | 258 |
|  | Superior Frontal Gyrus | R | 16 | 56 | -16 |  | 3.27 | 218 |
| PD-H > PD-L | Cingulate Gyrus | R | 24 | -22 | 30 |  | 4.55 | 882 |
|  | Posterior Cingulate | R | 8 | -40 | 22 |  | 3.95 | 882 |
|  | Tuber | R | 34 | -58 | -38 |  | 4.21 | 652 |
|  | Pyramis | L | -22 | -62 | -38 |  | 4.65 | 406 |
|  | Middle Temporal Gyrus | L | -32 | 6 | -40 |  | 4.41 | 382 |
|  | Medial Frontal Gyrus | R | 12 | 50 | -14 |  | 4.46 | 381 |

PD with the lowest SI volume group=PD-L; PD with the highest SI volume group=PD-H; R = right; L = left

**Supplementary Table 4**. Clusters displaying significantly different resting-state functional connectivity from seed region of interest in the posterior cingulate cortex.

|  | | **Brain Regions** | | **Side** | | **Stereotaxic Coordinates (mm)** | | | | |  | | **Maximum** | | | **Voxels** |
| --- | --- | --- | --- | --- | --- | --- | --- | --- | --- | --- | --- | --- | --- | --- | --- | --- |
|  | |  | |  | | **x** | **y** | | **z** | |  | | **t** | | |  |
| Control > PD-H | Lingual Gyrus | | L | | -22 | | | -50 | | 0 | |  | | 4.11 | 1180 | |
|  | Postcentral Gyrus | | R | | 42 | | | -30 | | 58 | |  | | 4.42 | 792 | |
|  | Lingual Gyrus | | R | | 12 | | | -64 | | 2 | |  | | 4.41 | 703 | |
|  | Precuneus | | R | | 24 | | | -68 | | 24 | |  | | 3.85 | 512 | |
|  | Precentral Gyrus | | L | | -54 | | | -10 | | 46 | |  | | 4.52 | 268 | |
|  | Postcentral Gyrus | | R | | 54 | | | -18 | | 46 | |  | | 3.68 | 235 | |
| Control > PD-L | Lingual Gyrus | | L | | -26 | | | -64 | | -6 | |  | | 3.75 | 714 | |
|  | Precuneus | | R | | 24 | | | -88 | | 50 | |  | | 4.05 | 555 | |
|  | Cuneus | | L | | -18 | | | -94 | | 28 | |  | | 4.34 | 262 | |
|  | Lingual Gyrus | | R | | 14 | | | -70 | | 0 | |  | | 3.26 | 247 | |
| PD-H > PD-L | Superior Frontal Gyrus | | L | | -18 | | | 56 | | 30 | |  | | 3.70 | 442 | |
|  | Parietal Lobule | | R | | 38 | | | -80 | | 52 | |  | | 3.44 | 397 | |
|  | Middle Temporal Gyrus | | R | | 64 | | | -30 | | -14 | |  | | 3.14 | 299 | |
|  | Superior Frontal Gyrus | | R | | 26 | | | 36 | | 58 | |  | | 3.11 | 236 | |

PD with the lowest SI volume group=PD-L; PD with the highest SI volume group=PD-H; R = right; L = left

**Supplementary Table 5**. Clusters displaying significantly different resting-state functional connectivity from seed region of interest in the substantia innominata.

|  | **Brain Regions** | **Side** | **Stereotaxic Coordinates (mm)** | | |  | **Maximum** | **Voxels** |
| --- | --- | --- | --- | --- | --- | --- | --- | --- |
|  |  |  | **x** | **y** | **z** |  | **t** |  |
| PD-H > Control | Declive | R | 10 | -76 | -18 |  | -4.72 | 1799 |
|  | Inferior Parietal Lobule | L | -38 | -68 | 44 |  | -3.15 | 959 |
|  | Declive | L | -24 | -74 | 72 |  | -3.75 | 959 |
|  | Inferior Frontal Gyrus | L | -40 | 2 | 28 |  | -3.76 | 645 |
|  | Pyramis | R | 24 | -62 | -42 |  | -3.74 | 543 |
| PD-L > Control | Lingual Gyrus | R | 14 | -88 | -24 |  | -4.41 | 1955 |
|  | Inferior Parietal lobule | L | -38 | -64 | 44 |  | -4.00 | 1426 |
|  | Inferior Frontal Gyrus | L | -56 | 28 | 16 |  | -4.38 | 570 |
|  | Superior parietal lobule | R | 32 | -80 | 44 |  | -3.98 | 479 |
|  | Tuber | R | 38 | -74 | -36 |  | -3.24 | 455 |
|  | Middle Frontal Gyrus | L | -50 | 56 | -6 |  | -4.25 | 311 |
|  | Superior Frontal Gyrus | L | -16 | 56 | 44 |  | -3.72 | 264 |
|  | Fusiform Gyrus | L | -50 | -42 | -18 |  | -3.97 | 245 |
|  | Precuneus | R | 20 | -56 | 52 |  | -4.16 | 238 |
| PD-H < PD-L | Inferior parietal lobule | R | 50 | -70 | 38 |  | -2.64 | 244 |

PD with the lowest SI volume group=PD-L; PD with the highest SI volume group=PD-H; R = right; L = left

**Supplementary Table 6**. Clusters displaying significant resting-state functional connectivity correlated with the SI volume with seed region of interest in the right caudate and posterior cingulate cortex. .

| **Seed** | **Brain Regions** | **Side** | **Stereotaxic Coordinates (mm)** | | |  | **Maximum** | **Voxels** |
| --- | --- | --- | --- | --- | --- | --- | --- | --- |
|  |  |  | **x** | **y** | **z** |  | **t** |  |
| Right caudate | Anterior Cingulate | R | 8 | 46 | 10 |  | 3.72 | 527 |
|  | Middle frontal gyrus | R | 20 | 34 | 48 |  | 3.49 | 509 |
|  | Superior frontal gyrus | L | -20 | 66 | 30 |  | 4.10 | 318 |
|  | Superior frontal gyrus | R | 4 | -8 | 68 |  | 3.66 | 266 |
|  | Parahippocampal gyrus | L | -28 | -20 | -26 |  | 3.39 | 244 |
| Posterior cingulate | Middle Temporal Gyrus | R | 64 | -30 | -16 |  | 3.93 | 328 |
|  | Superior Frontal Gyrus | R | 14 | 74 | 22 |  | 3.61 | 312 |
|  | Cuneus | R | 4 | -84 | 26 |  | 3.45 | 232 |

R = right; L = left

**Supplementary Table 7**. Clusters displaying significant resting-state functional connectivity correlated with the cognitive total composite score with seed region of interest in the caudate, posterior cingulate cortex and substantia innominata.

| **Seed** | **Brain Regions** | **Side** | **Stereotaxic**  **Coordinates (mm)** | | | **Maximum** | **Voxels** |
| --- | --- | --- | --- | --- | --- | --- | --- |
|  |  |  | **x** | **y** | **z** | **t** |  |
| Right Caudate | Inferior Parietal Lobule | L | -56 | -52 | 50 | 3.41 | 313 |
| Left caudate | Inferior Parietal Lobule | R | 54 | -54 | 38 | 4.85 | 713 |
|  | Tuber | L | -20 | -86 | -40 | 3.82 | 665 |
|  | Tuber | R | 26 | -80 | -40 | 3.60 | 414 |
|  | Inferior Parietal Lobule | L | -54 | -52 | 54 | 3.59 | 407 |
| Posterior cingulate | Superior Temporal gyrus | R | 66 | -30 | 8 | 4.32 | 1818 |
|  | Superior Temporal gyrus | L | -46 | -34 | 6 | 4.81 | 1774 |
|  | Paracentral Lobule | L | -16 | -42 | 50 | 3.93 | 538 |
|  | Precentral gyrus | R | 46 | -4 | 48 | 3.47 | 525 |
|  | Middle Frontal gyrus | L | -42 | -2 | 64 | 4.29 | 476 |
|  | Postcentral gyrus | R | 18 | -40 | 62 | 3.71 | 435 |
|  | Precuneus | R | 10 | -82 | 66 | 3.62 | 414 |
|  | Inferior Semi-Lunar Lobule | R | 18 | -66 | -50 | 3.77 | 375 |
|  | Paracentral Lobule | L | -4 | -16 | 86 | 3.97 | 268 |
|  | Middle Temporal gyrus | L | -32 | -64 | 10 | 3.61 | 262 |
| Substantia innominata | Cuneus | R | 18 | -110 | -8 | 5.13 | 2730 |
|  | Middle Frontal gyrus | R | 46 | 54 | 28 | 4.50 | 1223 |
|  | Lobule Vllb |  | -12 | -86 | -60 | 4.03 | 818 |
|  | Superior Frontal gyrus | R | 24 | 28 | 60 | 4.48 | 789 |
|  | Inferior Parietal Lobule | R | 62 | -24 | 30 | 3.80 | 650 |
|  | Cingulate gyrus | R | 8 | 32 | 32 | 4.69 | 572 |
|  | Cingulate gyrus | R | 26 | -40 | 44 | 4.21 | 336 |
|  | Transverse Temporal gyrus | L | -46 | -28 | 10 | 3.92 | 309 |
|  | Uvula | R | 28 | -96 | -38 | 3.59 | 303 |

R = right; L = left
